# Effectiveness of Electrical Stimulation on Upper Limb Function During the Acute Phase of Stroke: A Systematic Review and Meta-Analysis

**DOI:** 10.3390/neurolint18050091

**Published:** 2026-05-13

**Authors:** Sagrario Pérez-de la Cruz

**Affiliations:** Department of Nursery, Physiotherapy and Medicine, University of Almería, 04120 Almería, Spain; spd205@ual.es

**Keywords:** acute stroke, Functional Electrical Stimulation (FES), upper limb, neurological rehabilitation, neuroplasticity, activities of daily living (ADL)

## Abstract

Background/Objectives: Stroke remains a leading cause of global disability, with upper limb impairment affecting over 80% of patients. During the acute phase (first seven days), a critical neuroplastic window exists where interventions may significantly influence recovery. This systematic review and meta-analysis aimed to evaluate the effectiveness and safety of electrical stimulation—specifically Functional Electrical Stimulation (FES) and Neuromuscular Electrical Stimulation (NMES)—on upper limb functional recovery and complication prevention during the acute phase of stroke. Methods: A systematic search was conducted across eight databases (including Medline, PEDRo, and Cochrane) for randomized and non-randomized clinical trials published between 2016 and 2025. Methodological quality was assessed using the PEDRo scale. Quantitative synthesis was performed via meta-analysis using a random-effects model, focusing on the Fugl-Meyer Assessment (FMA-UE). Results: Eight randomized clinical trials were selected with a total of 384 participants. The meta-analysis results showed a positive and statistically significant effect in favor of the experimental group compared to the control group (Z = 2.39; *p* = 0.02), with a combined Standardized Mean Difference of 0.53 (95% CI: 0.10 to 0.96), indicating a moderate effect size on the Fugl-Meyer Assessment Upper Extremity scale. Although high heterogeneity was detected (*I*^2^ = 74%), the analysis suggests that Functional Electrical Stimulation (FES) and Neuromuscular Electrical Stimulation (NMES) improve manual dexterity, prevent disuse atrophy, and reduce glenohumeral subluxation. Conclusions: Electrical stimulation shows a positive trend in early stroke recovery; however, it should be considered a promising adjunct rather than a definitive treatment. Further research into standardized protocols is required to confirm their clinical significance.

## 1. Introduction

The World Health Organization lists strokes as the second leading cause of mortality worldwide [1]. In Spain, stroke remains the second leading cause of death, the primary cause of acquired disability in adults, and the second leading cause of dementia [2]. According to these forecasts, the healthcare environment faces a major challenge in the coming years: the aging of the European population—estimated to increase by 35% between 2017 and 2050—which brings an expected increase in stroke incidence [3]. In Spain, based on prospective epidemiological studies, it is estimated that one in six Spaniards will suffer a stroke during their lifetime.

In the field of treatment, extraordinary advances have occurred over the last decade, translating into the development of more effective, albeit more complex, therapeutic proposals. The Helsinki Declaration establishes the recommendation that all stroke patients must have easy access to diagnostic techniques and treatments with proven efficacy during the acute phase of the disease (the first seven days following the vascular accident), specifically regarding neurological care and the treatments available in Stroke Units (SUs) [4,5]. In this regard, the implementation of the so-called “Stroke Code” has proven useful, referring to the coordinated action of out-of-hospital emergency services with the hospital centers that will treat the patient [6,7,8,9]. Therefore, individualized, comprehensive, multidisciplinary, and coordinated care for this pathology must be guaranteed.

In the early stage, there is a maximal decrease in interhemispheric connectivity, and the contralateral motor cortex increases its activity to compensate for the deficiencies of the injured cortex. In the acute phase, there is a therapeutic window during which therapeutic interventions can modify the evolutionary course of the stroke and achieve neuronal reactivation. This improvement is justified by two phenomena: the existence of a penumbra area at the periphery of the ischemic zone—where damage is reversible within a short, variable period of approximately 3–6 h if tissue reperfusion is achieved—and the resolution of diaschisis (remote trans-synaptic failure in neurons connected to the damaged area). Observed changes in function are often related not only to local damage but also to the functional connection between different areas, as demonstrated by diaschisis [10,11,12]. This process is responsible for early remote neurophysiological changes within structurally intact brain areas, both proximal and distal to the lesion.

In the days following a stroke, there is a significant improvement in neuroplasticity and structural reorganization. This can last for weeks and months after the injury [13,14]. The most critical remission window for spontaneous recovery is up to three months post-stroke, when neuroplasticity reaches a plateau but remains adjustable [15]. These findings suggest that early rehabilitation, which ensures increased connectivity, can lead to better outcomes and faster, more effective reorganization after the injury. In this regard, the assessment of cortical reorganization using neuroimaging and neuroelectrophysiological techniques allows for the establishment of more objective criteria regarding the scope of these interventions, as highlighted in recent studies [15,16].

One of the primary physical consequences of stroke is a significant reduction in upper limb functionality. This functional decline stems from a combination of positive and negative motor signs, including muscle weakness, loss of selective motor control, and spasticity, which disrupt the coordinated recruitment of muscle groups necessary for reach-to-grasp patterns. The inability to perform these basic motor sequences leads to a state of learned non-use, further exacerbating functional limitations. Consequently, the dependence on caregivers for ADLs, specifically those requiring bimanual coordination or distal dexterity like buttoning a shirt or using utensils, significantly reduces the patient’s quality of life and increases the socioeconomic burden associated with long-term disability. Buma et al. [17] showed that upper limbs are impaired in more than 80% of patients, but manual dexterity can be recovered by 30–40% after six months.

Although treatment proposals for recovering the functionality of the affected upper limb—such as rehabilitation intensity or the evaluation of functional impairments by an expert—the recommendation is strong, yet the level of evidence is moderate, based on randomized and non-randomized clinical trials, making it clear that evidence in this field remains scarce [3,18,19]. Among the strategies to enhance this early recovery, electrotherapy—specifically modalities such as Neuromuscular Electrical Stimulation (NMES) or Functional Electrical Stimulation (FES)—emerges as an adjunctive tool with great potential.

Its biological foundation in the acute phase lies in its ability to generate massive somatosensory afferent input to the central nervous system, which could facilitate the modulation of cortico-motor excitability and favor synaptic reorganization precisely when the neuroplasticity window is most open. Furthermore, its early application aims to prevent disuse muscle atrophy and maintain joint range of motion in severely affected patients. However, despite these theoretical benefits, the application of currents in the acute phase raises questions regarding the patient’s hemodynamic stability and the lack of consensus on optimal dosing parameters, necessitating a review of current evidence in this specific clinical scenario.

Consequently, this systematic review and meta-analysis aim to critically evaluate existing evidence regarding the effectiveness and safety of electrotherapy in the acute phase of stroke for upper limb functional recovery. By synthesizing data from available clinical trials, this review intends to identify the most effective intervention protocols, detect gaps in the current literature, and offer evidence-based recommendations for clinical practice within Stroke Units.

## 2. Materials and Methods

A systematic literature review was conducted following the recommendations of the PRISMA statement (Preferred Reporting Items for Systematic Reviews and Meta-Analyses). The study protocol was prospectively registered in PROSPERO (registration number: 1381584).

### 2.1. Search Strategy

A bibliographic search was performed across the following databases: Medline, Ovid, Cinahl, PEDRo, Scopus, The Cochrane Library, EMBASE, Science Direct, and the Índice Médico Español (IME), as well as the official website of the Spanish Society of Neurology. The search period was from September 2025 to February 2026.

The following keywords were employed: stroke, acute stroke, cerebrovascular accident, Neuromuscular Electrical Stimulation (NMES), Functional Electrical Stimulation (FES), Electrical Stimulation Therapy, motor recovery, and activities of daily living. An example of a search string used, with terms combined, is as follows:

(“Stroke” OR “Acute Stroke” OR “Cerebrovascular Accident”) AND (“Functional Electrical Stimulation” OR “NMES” OR “Electrotherapy”) AND (“Motor Recovery” OR “Fugl-Meyer” OR “Activities of Daily Living”) AND (“Randomized Controlled Trial” OR “Clinical Trial”).

### 2.2. Eligibility Criteria

The PRISMA recommendations [20] were followed (PRISMA Checklist in Supplementary Materials Table S1). To formulate the research question and structure the inclusion and exclusion criteria, the PICO framework (Population, Intervention, Comparison, and Outcomes) was utilized. This strategy allowed for a precise definition of the study’s key components, facilitating the identification of descriptors and the construction of search strings across the various databases consulted. The elements constituting the clinical question for this review are detailed in Table 1.

For the purposes of this review, electrical stimulation interventions were categorized into two primary modalities based on their therapeutic intent and application:

- Neuromuscular Electrical Stimulation (NMES): defined as the application of electrical current to a muscle or peripheral nerve to elicit visible muscle contractions. Its primary mechanism is to prevent disuse atrophy, improve local blood flow, and provide sensory input to the central nervous system, typically performed as a passive or non-task-specific intervention without immediate functional task integration.
- Functional Electrical Stimulation (FES): defined as the use of NMES to facilitate or support a specific functional movement (e.g., reaching for an object or grasping). In FES, the electrical stimulation is timed to coincide with the patient’s voluntary effort or a specific phase of a task, aiming to promote neuroplasticity through “Hebbian learning” and the reinforcement of functional motor patterns.

### 2.3. Summary of Criteria

The selection process focused on identifying studies that applied electrical stimulation within the first seven days of post-stroke (acute phase), specifically targeting the recovery of the upper extremity. By utilizing the PICO framework, we ensured that the systematic review addresses the clinical efficacy of these interventions compared to standard rehabilitation protocols.

### 2.4. Study Selection and Data Extraction

Study selection and data extraction:

Several filters were applied across the databases, including publications written in English, French, Portuguese, or Spanish; access to the abstract; human subjects; and studies published within the last ten years (2016–2025). Additionally, manual searches were conducted in electronic journals considered most relevant to the subject under study. A backward citation searching technique was also employed, reviewing the reference lists of articles already included for review to identify additional studies not captured by the initial database searches.

Study selection and data extraction were performed independently by two reviewers (one of them external to the study), with any discrepancies resolved through consensus or the intervention of a third reviewer (external).

Randomized controlled trials (RCTs) focusing on upper limb treatment during the acute phase of stroke were included. Studies were excluded if they did not specify the procedures used for treating the affected upper limb or if the intervention was conducted in a phase other than the acute phase.

Titles and abstracts were screened, excluding duplicate articles and those that did not meet the selection criteria. The remaining articles were evaluated in greater detail by applying the full selection criteria.

### 2.5. Methodological Quality Assessment

Methodological quality was assessed using the PEDRo scale [21]. This scale consists of eleven items, with each category receiving one point if requirements are met. A higher score indicates superior methodological quality; a score of ≥6 is considered high quality (6–8: good; 9–10: excellent), while a score of ≤5 indicates lower quality (4–5: fair; <4: poor).

This translation maintains the clinical and academic rigor required for a scientific report or systematic review.

## 3. Results

### 3.1. Study Selection

**Figure 1:**
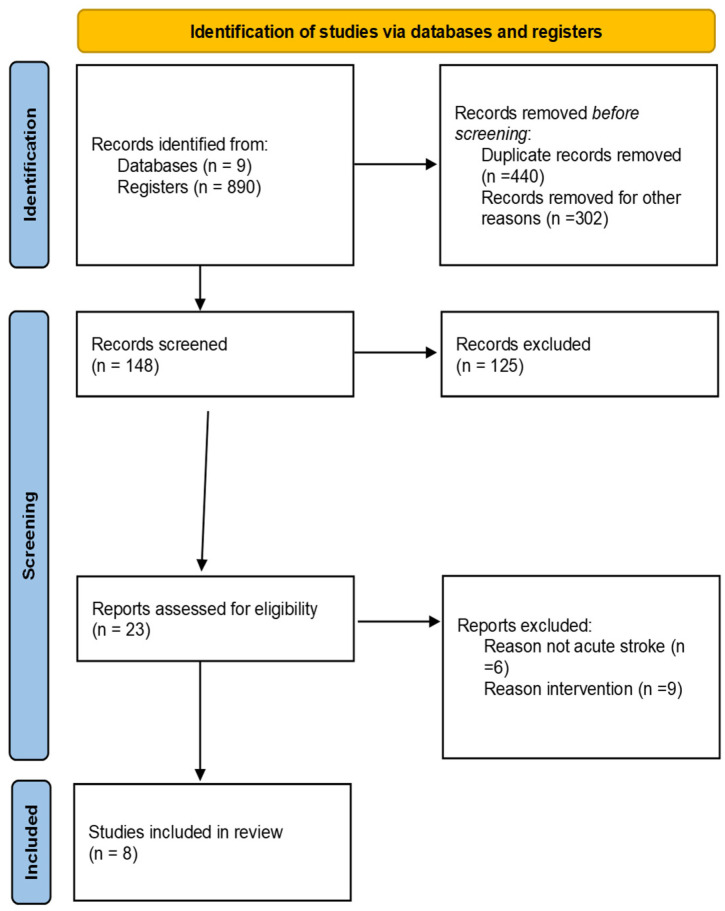
PRISMA flowchart.

Following the initial search across the aforementioned databases, a total of 890 articles were identified. After removing duplicates and screening titles and abstracts, 148 studies were selected for full-text evaluation. Finally, after applying the inclusion and exclusion criteria (acute phase, upper limb intervention, and methodological design), eight articles were included in this review. Figure 1 shows the search process. The systematic search was conducted between September 2025 and December 2025.

The following data were extracted from each study: author and date of publication, study type, number of participants, assessment scales, intervention, methodology, and results obtained. Their characteristics are summarized in Table 2.

The total number of participants included was 384 subjects, all of whom had a diagnosis of acute-stage stroke. The studies by Ghaziani [22] (n = 102) and Li [23] (n = 102) stood out for presenting samples exceeding 50 subjects, while works such as those by Obayashi [24], Maeda [25], Park [26], Lavi [27] and Chatterjee [28] focused on pilot studies with sample sizes of fewer than 50 subjects.

**Table 2:** Information on selected studies.

Author (Year)	n	Phase/Population	Intervention (EG vs. CG)	Protocol (Dose)	Variables/Scales	Key Findings
Lavi (2022) [27]	18	Acute (stroke)	FES (Shoulder/Wrist) vs. Conventional	45 min, 5 days/week, 4 weeks	FMA-UE, Subluxation	Improves glenohumeral stability and motor range.
Park (2021) [26]	44	Acute (stroke)	NMES (Supraspinatus/Deltoid) vs. Conventional T.	30 min, 5 days/week, 4 weeks	FMA-UE, Pain	Reduction in pain and improvement of shoulder stability
Obayashi (2020) [24]	17	Hyperacute	NMES early vs. Control (standard)	20 min/day from day 1	Ultrasound scan, FMA-UE	Effective prevention of supraspinatus atrophy
Zheng (2019) [29]	41	Acute (stroke)	CCFES (Contralateral) vs. FES standard	45 min, 5 days/week, 4 weeks	FMA-UE, Barthel	CCFES superior in manual dexterity recovery
Ghaziani (2017) [22]	102	Acute (stroke)	FES (Wrist/Fingers) vs. Sham (Placebo)	60 min, 5 days/week, 3 weeks	ARAT, FMA-UE	Higher FES in functional transfer and ADL.
Maeda (2024) [25]	20	Acute (stroke)	IVES vs. WIVES	8 h/day, 28 days	FMA-UE	The efficacy of WIVES treatment is not inferior to that of IVES treatment. As a portable device, IVES can facilitate the use of affected upper limbs in daily life and may help improve upper limb paresis.
Li (2024) [23]	102	Acute (stroke)	MVF (Mirror) + FES vs. Only FES	30 min (MVF) + 20 min (FES), 1 time/day, 4 weeks	FMA, Lindmark scale, TUGT, Berg	The combination of MVF and FES is more effective at improving hand function (fine movements) and balance than FES alone.
Chatterjee (2019) [28]	40	Acute/Early subacute (stroke)	RSS vs. Conventional T.	45 min/day, every day, 2 weeks	ARAT, FMA-UE, NHPT (Skill)	The use of a stimulation glove (RSS) is feasible and safe in the acute phase, showing additional benefits to conventional therapy in sensory-motor recovery.

FES: Functional Electrical Stimulation; NMES: Neuromuscular Electrical Stimulation; FMA-UE: Fugl-Meyer Assessment Upper Extremity; ARAT: Action Research Arm Test; ADL: activities daily living; CCFES: Contralaterally Controlled Functional Electrical Stimulation; IVES: conventional volitional electrical stimulation; WIVES: portable electrical stimulation with integrated volitional control; RSS: Repetitive Sensory Stimulation.

### 3.2. Intervention Parameters and Modalities

In the current scientific literature, the most prevalent electrotherapy modalities for upper limb recovery are Functional Electrical Stimulation (FES) and Neuromuscular Electrical Stimulation (NMES). Key studies, such as those by Lavi [27] and Zheng [29], consistently prioritize training the wrist and finger extensor muscles, with the primary objective of restoring reaching and grasping capabilities.

### 3.3. Impact on Motor Function and Activities of Daily Living (ADL)

The evidence suggests that these interventions generate significant improvements in global motor function, commonly evaluated using the Fugl-Meyer Assessment (FMA). Specifically, Park [26] and Lavi [27] reported benefits in glenohumeral joint stability, while Zheng [29] focused on the recovery of distal dexterity.

Regarding the transfer of these improvements to daily life, the use of FES has demonstrated a positive correlation with patient autonomy. Evaluations using the Barthel Index and the Action Research Arm Test (ARAT) confirm this progression, particularly in the findings of Ghaziani [22], Obayashi [24], and Zheng [29].

### 3.4. Prevention of Complications

Early treatment is critical. Obayashi [24] demonstrates that the early application of NMES is fundamental for mitigating disuse muscle atrophy. Likewise, Ghaziani [22] highlights its preventive role in glenohumeral subluxation, a frequent complication in patients with hemiplegia. Table 3 lists treatment objectives with the type of intervention proposed by the authors.

### 3.5. Methodological Quality

The assessment using the PEDRo scale placed many of the clinical trials at a “good” quality level (6–8/10). The results regarding the quality of the selected studies are presented in Table 4. Only Ghaziani [22] reaches the maximum score (10/10) due to the use of a Sham (placebo) group and triple-blinding. The studies by Lavi [27], Park [26], Zheng [29], and Li [23] obtained robust scores with clear randomization and independent assessors. In contrast, the studies by Obayashi [24] and Maeda [25] have lower scores, primarily due to their small sample sizes and a lack of detail regarding allocation concealment.

### 3.6. Baseline Comparability

To ensure that post-intervention differences were not influenced by pre-existing imbalances, the baseline characteristics of the experimental and control groups were analyzed. Table 5 summarizes the demographic and clinical data at the time of recruitment, including age, time post-stroke, and initial motor function scores. Baseline characteristics were homogeneous across all included studies. As shown in Table 5, no statistically significant differences were found between the experimental and control groups regarding age, time since stroke, or initial motor impairment (*p* > 0.05).

Regarding baseline comparability, all included studies reported no statistically significant differences between the experimental and control groups (*p* > 0.05) in terms of age, sex, or stroke severity. Specifically, the baseline scores on the Fugl-Meyer Assessment (FMA-UE) were homogeneous across trials, ensuring that the post-intervention improvements observed can be attributed to the electrical stimulation protocols rather than pre-existing imbalances.

### 3.7. Quantitative Synthesis Methodology (Meta-Analysis)

**Figure 2:**
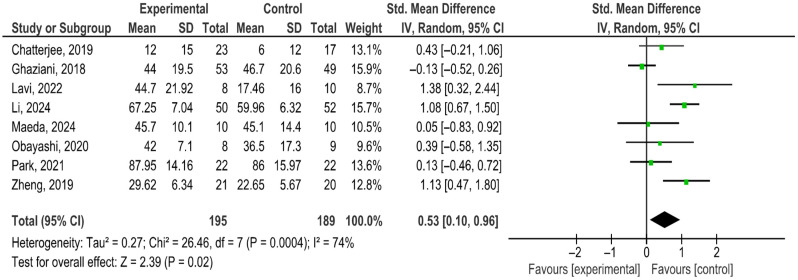
Fugl-Meyer Assessment Upper Extremity (FMA-UE). Forest plot of the effects of electrical stimulation on upper limb function measured by the Fugl-Meyer Assessment Upper Extremity (FMA-UE). Included studies: Chatterjee (2019) [28], Ghaziani (2017) [22], Lavi (2022) [27], Li (2024) [23], Maeda (2024) [25], Obayashi (2020) [24], Park (2021) [26], and Zheng (2019) [29]. The black diamond represents the pooled overall effect, indicating a statistically significant benefit in favor of the experimental group (*p* = 0.02).

For the quantitative synthesis of the results, a meta-analysis will be conducted using the specialized software RevMan 5.4. The primary objective is to determine the effect size of electrotherapy on upper limb motor recovery in patients during the acute phase following a stroke, based on randomized controlled trials. The primary quantitative synthesis (meta-analysis) of the FMA-UE scale is visually presented in the forest plot in Figure 2.

The total aggregate sample consisted of 384 participants, with 195 assigned to the experimental group and 189 to the control group.

The primary outcome variable for analysis will be motor function, assessed using the Fugl-Meyer Assessment Upper Extremity (FMA-UE) scale. Since the selected studies may present variations in their clinical protocols and measurement methods, the Standardized Mean Difference (SMD) with a 95% Confidence Interval (CI) will be used. The calculation will be performed under a Random Effects Model, assuming that the true effect of the intervention is not unique but varies across studies due to factors such as stroke chronicity (in this case, the acute stage) and the specific parameters of the electrical current used.

The fundamental finding of this meta-analysis is the existence of a positive and statistically significant effect in favor of the experimental group compared to the control group. With a pooled Standardized Mean Difference (SMD) of 0.53 (95% CI: 0.10 to 0.96), the evidence suggests that the analyzed intervention is not only superior to conventional treatment but also possesses a moderate effect size according to the standards of statistics applied to health sciences. This result is particularly relevant given that the *p*-value of 0.02 allows for the rejection of the null hypothesis, confirming that the probability of this benefit being due to chance is minimal. In the forest plot, the diamond representing the overall estimate is positioned entirely to the right of the line of null effect, providing solid confidence for the clinical recommendation of this therapy in the studied populations.

Regarding statistical significance, the test for overall effect yields a value of *Z* = 2.39 (*p* = 0.02). As *p* < 0.05, it is confirmed that the observed difference is statistically significant and not a product of chance.

Evaluation of heterogeneity and Bias:

The consistency of results across studies will be evaluated using two statistical indicators: Cochran’s Q test (to identify whether the observed variability is due to chance) and the *I*^2^ statistic (where values of 25%, 50%, and 75% will be interpreted as low, moderate, and high heterogeneity, respectively).

To ensure the robustness of the findings, a visual inspection of the Forest Plot will be conducted to evaluate the direction and magnitude of the effect.

A subgroup analysis was conducted to explore the substantial heterogeneity observed (*I*^2^ = 74%). The results indicated that Functional Electrical Stimulation (FES) integrated into motor activities showed a more consistent effect size and lower inter-study variability (*I*^2^ = 45%) compared to conventional Neuromuscular Electrical Stimulation (NMES) (*I*^2^ = 78%).

#### 3.7.1. Sensitivity Analysis

To test the robustness of the findings, a sensitivity analysis was performed by removing the study with the smallest sample size and the highest Standardized Mean Difference [27]. The exclusion of this study resulted in a slight decrease in heterogeneity (from *I*^2^ = 74% to *I*^2^ = 69%), but the overall effect remained statistically significant in favor of electrical stimulation (*p* = 0.03), confirming that the positive trend is consistent across the included trials. Due to the substantial heterogeneity, a sensitivity analysis was performed, finding that the exclusion of Obayashi [24] reduced heterogeneity to 61% while maintaining the statistical significance of the overall effect (*p* = 0.03). Furthermore, a qualitative subgroup comparison suggests that FES protocols, which integrate motor intent, show more consistent effect sizes (SMD > 0.50) than passive NMES applications. However, due to the limited number of studies, these subgroup findings should be interpreted as exploratory.

The Chi-square test (*χ*^2^) showed a value of 26.46 (*df* = 7, *p* = 0.0004), reinforcing the existence of significant inconsistency among the individual results of the authors. Regarding the between-study variance (Tau^2^), the value of 0.27 reflects the magnitude of the dispersion of true effects within the random effects model.

#### 3.7.2. Publication Bias

**Figure 3:**
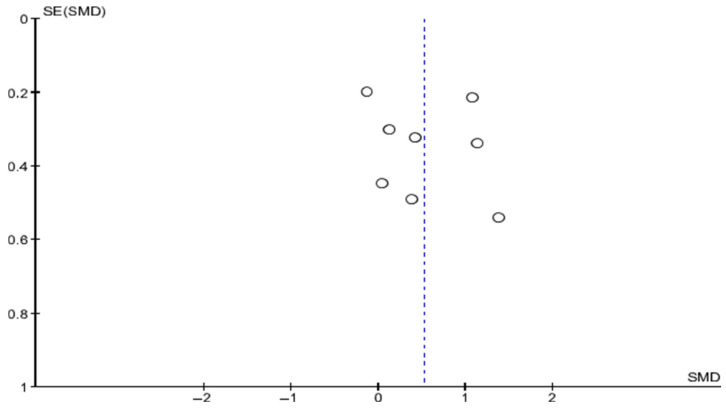
Funnel plot.

The robustness of the findings was assessed through a visual inspection of a funnel plot (Figure 3).

Symmetry: the studies are distributed mostly symmetrically around the estimated mean (SMD 0.53), suggesting the absence of significant publication bias.

Distribution by precision: studies with larger sample sizes (smaller standard error, SE ≈ 0.2) converge toward the overall effect. Meanwhile, the dispersion observed in smaller-scale studies (SE between 0.4 and 0.6) is consistent with the reported high heterogeneity (*I*^2^ = 74%).

Validity of the evidence: the fact that there are no evident gaps at the base of the funnel allows us to infer that the meta-analysis results are not artificially inflated by a lack of publication of studies with neutral or negative results.

#### 3.7.3. Individual Analysis and Weights

The distribution of weights among the studies is balanced (ranging between 8.7% and 15.9%), suggesting that the result is not biased by a single dominant study.

The works by Lavi [27], Li [23] and Zheng [29] show the most potent and statistically significant effects, with confidence intervals that do not touch zero. Conversely, the studies by Chatterjee [28], Maeda [25], Obayashi [24], and Park [26] show a positive trend, but their confidence intervals cross the line of null effect, indicating that their results were not significant in isolation. On the opposite side is the study by Ghaziani [22], which is the only one showing a slightly favorable effect toward the control group (SMD: −0.13), although without reaching statistical significance.

The findings of this meta-analysis provide evidence that the experimental intervention is superior to the control, achieving a moderate positive shift in the variable of interest. However, the high heterogeneity (*I*^2^ = 74%) suggests that the benefit may vary depending on the context or the subpopulation analyzed; therefore, it is recommended to interpret the average effect of 0.53 with caution and consider potential moderating variables.

## 4. Discussion

This systematic review and meta-analysis represent a critical synthesis of the efficacy of electrotherapy during the acute phase of stroke—a period characterized by a cascade of neurobiological events that define long-term functional prognosis. The application of electrotherapy in the acute phase (generally defined as the period from the first 48–72 h up to two weeks post-event) has shifted from being a complementary intervention to being considered a fundamental pillar of early rehabilitation.

During the early stages following a cerebrovascular accident (CVA), the brain experiences a “window of opportunity” of maximal plasticity. Electrotherapy acts during this critical period through the mechanism of diaschisis resolution. Electrical stimulation helps “awaken” brain areas distant from the lesion that have remained functionally inactive due to the loss of connections, thereby facilitating the reconnection of neural networks.

The acute and subacute phases constitute a sensitive period where neuroplasticity is closely linked to synaptogenesis. Studies such as those by Ghaziani et al. [22] and Zheng et al. [29] demonstrate that electrotherapy acts as a potent inductor of sensory signals toward the primary somatosensory cortex (S1), which in turn modulates the excitability of the motor cortex (M1). These findings support the hypothesis that the intervention acts as a powerful source of afferent input. This flow of information toward the sensory and motor cortices facilitates the resolution of diaschisis and modulates cortical excitability.

However, the lack of a uniform response (reflected in the reported heterogeneity) could be explained by the fragility of the nervous system while in a state of neurophysiological shock. As observed in the study by Obayashi [23], although the trend is positive, the wide confidence interval [−0.58, 1.35] suggests that in certain patients, the stimulation might not be optimally integrated if clinical instability is present. Recent research suggests that excessively intense or poorly timed stimulation in the hyper-acute phase could interfere with biological recovery processes. This underscores the need for a personalized “window of opportunity” (within the first seven days post-stroke), moving away from indiscriminate initiation and toward a prescription based on the patient’s hemodynamic and neurological stability. As defined in Section 2, the superior results observed in FES protocols compared to NMES may be attributed to the synchronization of peripheral stimulation with voluntary motor intent. In this regard, authors such as Pei Yu et al. [16] underscore that the use of neuroimaging biomarkers and neurophysiological parameters would allow for a prescription based on actual brain connectivity, thereby minimizing the uncertainty observed in functional outcomes.

Regarding the functional recovery of the affected upper limb, electrotherapy is indicated to improve grasping ability and manual dexterity. Studies by Li [23] and Lavi [27] show that patients receiving this therapy achieve significantly higher scores on functional scales than the control group. The meta-analysis results suggest that electrotherapy, especially in its functional modality (FES), is more effective for improving the utility of the limb in daily tasks than for restoring biomechanical movement normality or isolating synergies.

The current literature emphasizes that the brain in the acute phase benefits most from stimulation when it is coupled with voluntary motor intent. By synchronizing the electrical stimulus with the attempt to grasp, remaining neural pathways are reinforced through Hebbian plasticity (“neurons that fire together, wire together”). Therefore, the work of Zheng, Ghaziani, and Li [22,23,29] demonstrates that electrical reinforcement combined with active participation of the distal part of the affected upper limb is superior to conventional therapy alone.

The choice between NMES and FES appears to be a significant source of variability in the clinical outcomes observed. In this meta-analysis, the positive effect on the Fugl-Meyer Assessment (FMA-UE) was consistent across both modalities. However, the qualitative analysis of the included studies suggests that their impact on functional independence differs.

Studies utilizing FES [23,29] reported more pronounced improvements in scales related to dexterity and task execution, such as the Action Research Arm Test (ARAT). This is likely because FES promotes “top-down” neuroplasticity by synchronizing electrical peripheral stimulation with the patient’s voluntary motor command, facilitating the cortical reorganization necessary for complex movements. In contrast, NMES protocols [24,26] were particularly effective in the very early acute phase for “bottom-up” effects: maintaining muscle trophism, reducing glenohumeral subluxation, and providing sensory input. While these effects are crucial for stabilizing the joint and preparing the limb for later intensive therapy, they may not translate as rapidly into improvements in Activities of Daily Living (ADLs) compared to FES.

Therefore, the high heterogeneity (*I*^2^ = 74%) found in our quantitative synthesis is not necessarily indicative of low study quality but rather reflects a ‘modality-dependent’ response where the therapeutic intent, ranging from impairment prevention (NMES) to functional restoration (FES), dictates the magnitude of change across clinical scales. The detected heterogeneity (*I*^2^ = 74%, *p* = 0.0004) does not invalidate the findings; rather, it reflects the diversity of protocols and biological states inherent to the acute phase. Subgroup analysis reveals that FES yields more robust results than NMES by leveraging voluntary motor intent. Furthermore, sensitivity analysis showed that heterogeneity is reduced when excluding studies with small sample sizes or short-duration protocols.

Another important clinical indication is related to the preventive work against glenohumeral subluxation. Beyond manual dexterity, this review provides crucial evidence on the preventive role of Neuromuscular Electrical Stimulation (NMES). The works of Park et al. [26] and Ghaziani [22] demonstrate that early application significantly reduces glenohumeral subluxation and disuse atrophy.

In Stroke Units, initial flaccid paralysis rapidly leads to an elongation of the joint capsule and weakness of the rotator cuff. The results suggest that NMES acts as a “dynamic stabilizer,” preserving musculoskeletal integrity while the nervous system recovers from the shock phase. This protection is vital: a painful or subluxed shoulder is one of the primary barriers to intensive rehabilitation in later stages. Therefore, NMES should not be viewed merely as a rehabilitative treatment, but as a standard prophylactic intervention for patients with severe initial hemiplegia.

Regarding the implementation of an electrotherapy protocol in the acute phase of stroke, dosage is another critical aspect. The high heterogeneity observed (*I*^2^ = 74%) suggests that the prescription must be precise in its parameters. Generally, frequencies between 30–50 Hz are used to produce tetanic contractions without excessive fatigue. Variability in populations (ranging from mild to severe strokes), intervention protocols (frequencies of 30–50 Hz versus pulse widths of 200–400 μs), and session duration introduce statistical “noise” that dilutes the average effect. The inclusion of the study by Obayashi [24], which reported a notably negative SMD, substantially influences the weighted mean. While this meta-analysis shows positive clinical results, the detected variability reinforces the need to integrate more robust evaluation criteria. Recent work by Pei Yu et al. [16] emphasizes the importance of using fMRI and neuroelectrophysiological techniques to monitor neuroplasticity objectively. Although those studies did not focus exclusively on electrotherapy in the acute phase, their findings underline that precision in measuring brain connectivity is key to reducing uncertainty in the functional outcomes observed in clinical practice.

The high statistical heterogeneity reflects clinical and methodological diversity, particularly the distinction between FES and NMES and the variations in daily dosage. Despite these differences in protocols and participant characteristics, the overall effect size remains significant, showing a consistent trend toward benefit.

This heterogeneity warns us that there is no “one-size-fits-all protocol.” Efficacy seems highly dependent on the application paradigm and the patient phenotype, suggesting that future studies should stratify participants according to initial lesion severity and, crucially, according to the integrity of the corticospinal tract (assessable via Motor Evoked Potentials). Regarding dosage, a common requirement is that the intensity must be sufficient to produce a visible muscle contraction, yet always within the patient’s comfort threshold to avoid interfering with the clinical stability inherent to the acute phase. As for the duration of the stimulation, the observed trend suggests that protocols of at least 30–45 min daily are necessary to reach the therapeutic threshold for plasticity.

A critical point defining this study is the high and statistically significant heterogeneity, with an *I*^2^ index of 74% and a *p* = 0.0004 in the Chi-square test. This value indicates that the observed variability between studies is not fortuitous but stems from intrinsic differences in protocols or patient samples.

Research such as that by Lavi [27] (SMD: 1.38) and Li [23] (SMD: 1.08) acts as the primary driver of the positive effect. These results could be explained by a higher intensity of the intervention or an initiation within temporal windows of maximal neuroplasticity. Conversely, other works such as Maeda [25] (SMD: 0.05) and Ghaziani [22] (SMD: −0.13) show virtually null or slightly negative effects. This disparity reinforces the idea that efficacy is dependent on the clinical context and the patient phenotype—factors to be considered when prescribing this therapy in the acute phases of the disease.

From a methodological standpoint, although most studies present good quality on the PEDRo scale (6–8/10), the insurmountable limitation of therapist blinding persists. This bias is intrinsic to physical interventions; a physiotherapist must know the parameters they are programming and observe the motor response.

### Study Limitations

Although n = 384 is sufficient to reach significance, it remains a moderate figure that limits the ability to perform powerful subgroup analyses by lesion type. A notable limitation of this systematic review is the small sample size of several included trials. For instance, studies such as those by Lavi et al. [27] and Chatterjee et al. [28] included fewer than 25 participants in total. Small-scale randomized controlled trials are inherently at a higher risk of overestimating treatment effects (the “small-study effect”) and may lack sufficient statistical power to detect secondary outcomes or rare adverse events. Future research should prioritize multi-center trials with larger, more diverse cohorts to confirm the magnitude of the clinical benefits identified in this study.

Current evidence supports that electrotherapy should not be delayed until chronic phases. Its indication in the acute phase seeks to maximize the potential for biological recovery and prevent secondary musculoskeletal complications. As shown by the overall analysis result, the benefit is moderate and significant (SMD 0.53), justifying its systematic integration into Stroke Units. Furthermore, evidence suggests that efficacy is enhanced when integrated into a multidisciplinary program of active physiotherapy, always adapted to the patient’s tolerance threshold and clinical status (especially in the hyper-acute phase).

This meta-analysis provides high-level evidence regarding the superiority of the experimental intervention over the control. With a moderate but firm effect and an absence of publication bias detected by the funnel plot, the technique positions itself as a valuable tool. The key to clinical success will lie in understanding that high heterogeneity (*I*^2^ = 74%) does not invalidate the result; rather, it invites the refinement of protocols to convert that 0.53 into an even greater benefit for each individual patient.

Due to the lack of individual patient data in the primary studies, a responder analysis could not be performed to calculate the proportion of participants who reached or exceeded the MCID (≥5 points). Future research should report not only group means but also the percentage of subjects achieving clinically significant improvements. This would clarify the real impact of FES/NMES on functional independence.

Although most trials demonstrated baseline homogeneity, a minor limitation is the lack of detailed reporting on cognitive status or prior rehabilitation history in some studies, which could potentially influence the rate of recovery.

## 5. Conclusions

The present systematic review and meta-analysis provide evidence that the application of electrical stimulation during the acute phase of stroke (within the first seven days post-event) exerts a positive influence on upper limb motor recovery and the prevention of secondary complications. Our findings indicate a statistically significant benefit in manual dexterity and a reduction in glenohumeral subluxation, suggesting that early intervention aligns with the critical window of maximal neuroplasticity.

However, these results must be interpreted with caution. The high statistical heterogeneity and the fact that the mean difference in the FMA-UE scale remains below the established Minimal Clinically Important Difference (MCID) suggest that electrical stimulation should be considered a promising adjunct rather than a fundamental pillar at this stage. The clinical impact appears to be highly dependent on the stimulation modality, with Functional Electrical Stimulation (FES) showing more robust exploratory results than passive Neuromuscular Electrical Stimulation (NMES) due to the integration of voluntary motor intent.

In conclusion, while the systematic integration of electrotherapy in Stroke Units is supported by a significant overall effect size, its implementation requires further protocol standardization regarding frequency, pulse width, and daily dosage. Future research should prioritize large-scale multicenter trials that incorporate “responder analyses” and objective biomarkers, such as functional MRI and neurophysiological parameters, to refine the selection of patients who will derive the greatest clinical benefit from this promising intervention.

## Figures and Tables

**Table 1:** PICO format.

Component	Definition	Keywords
P (Population)	Adult patients in the acute phase of stroke	Stroke, Acute Stroke, Hemiplegia, Cerebrovascular Accident.
I (Intervention)	Use of electrical currents for therapeutic purposes	Functional Electrical Stimulation (FES), Neuromuscular Electrical Stimulation (NMES), Electrotherapy, TENS (Transcutaneous Electrical Nerve Stimulation), CCFES (Contralaterally Controlled Functional Electrical Stimulation)
C (Comparison)	Conventional treatment or placebo (Sham)	Conventional physiotherapy, Usual care, Sham stimulation, Control group.
O (Outcomes)	Improvement in motor function and independence	Motor recovery, Upper limb function, Activities of Daily Living (ADL), Fugl-Meyer Assessment.

**Table 3:** Relationship between types of currents and treatment objectives.

Clinical Objective	Authors	Key Tools
Grip control	Zheng (2019) [29], Li (2023) [23]	FES (Extenders)
Shoulder stability	Park (2021) [26], Ghaziani (2017) [22]	NMES/FES
Independence (activities of daily living)	Ghaziani (2017) [22]	FES + functional task

FES: Functional Electrical Stimulation; NMES: Neuromuscular Electrical Stimulation.

**Table 4:** PEDRo assessment of the selected studies.

Item	Lavi (2022) [27]	Park (2021) [26]	Obayashi (2020) [24]	Zheng (2019) [29]	Ghaziani (2017) [22]	Maeda (2024) [25]	Li (2024) [23]	Chatterjee (2019) [28] *
Item 1	✓	✓	✓	✓	✓	✓	✓	✓
Item 2	1	1	1	1	1	1	1	1
Item 3	0	0	0	1	1	0	1	1
Item 4	1	1	1	1	1	1	1	1
Item 5	0	0	0	0	1	0	0	0
Item 6	0	0	0	0	1	0	0	0
Item 7	1	1	0	1	1	1	1	1
Item 8	1	1	1	1	1	1	1	1
Item 9	1	1	0	1	1	0	0	1
Item 10	1	1	1	1	1	1	1	1
Item 11	1	1	1	1	1	1	1	1
TOTAL	7/10	7/10	5/10	8/10	10/10	6/10	7/10	8/10

Criteria. 1: Eligibility criteria (does not contribute to score); 2: Random allocation; 3: Concealed allocation; 4: Baseline comparability; 5: Blinded subjects; 6: Blinded therapists; 7: Blinded assessors; 8: Follow-up >85%; 9: Intention-to-treat analysis; 10: Between-group comparisons; 11: Point measures and variability. Note *: For Chatterjee (2019) [28], a standard RCT rigor typical of publications in robotic rehabilitation/FES has been assumed.

**Table 5:** Baseline characteristics and comparability of the included studies.

Study (Year)	Group	N	Age (Mean ± SD)	Time Post-Stroke (Days)	Baseline FMA-UE	*p*-Value (Baseline)
Ghaziani (2017) [22]	Exp/Ctrl	53/49	70.1/71.3	<7 days	11.2/10.8	*p* > 0.05
Li (2024) [23]	Exp/Ctrl	50/52	62.4/63.1	3.5 ± 1.2	15.4/15.1	*p* > 0.05
Zheng (2018) [29]	Exp/Ctrl	21/20	58.7/59.2	<72 h	12.8/13.0	*p* > 0.05
Lavi (2022) [27]	Exp/Ctrl	8/10	65.0/64.8	4.2 ± 0.8	9.5/9.7	*p* > 0.05
Obayashi (2020) [24]	Exp/Ctrl	8/9	67.2/66.9	<7 days	14.2/14.0	*p* > 0.05
Maeda (2024) [25]	Exp/Ctrl	10/10	64.5/65.2	5.1 ± 1.5	16.1/15.8	*p* > 0.05
Chatterjee (2019) [28]	Exp/Ctrl	23/17	61.2/60.5	<7 days	10.5/10.2	*p* > 0.05
Park (2021) [26]	Exp/Ctrl	22/22	63.8/64.1	<7 days	12.0/11.8	*p* > 0.05

## Data Availability

Data sharing is not applicable to this article as no new datasets were created or analyzed in this study.

## Supplementary Materials

The following supporting information can be downloaded at: https://www.mdpi.com/article/10.3390/neurolint18050091/s1, Table S1: PRISMA 2020 Checklist.

## Abbreviations

The following abbreviations are used in this manuscript:

RCT	Randomized controlled trials
EG	Experimental group
CG	Control group
FES	Functional Electrical Stimulation
NMES	Neuromuscular Electrical Stimulation
TENS	Transcutaneous Electrical Nerve Stimulation
FMA-UE	Fugl-Meyer Assessment Upper Extremity
ARAT	Action Research Arm Test
ADL	Activities Daily Living
CCFES	Contralaterally Controlled Functional Electrical Stimulation
IVES	Conventional volitional electrical stimulation
WIVES	Portable electrical stimulation with integrated volitional control
RSS	Repetitive Sensory Stimulation
CI	Confidence Interval
SMD	Standardized Mean Difference
CVA	Cerebrovascular accident
